# Medium and long-term prognosis in hospitalised older adults with multimorbidity. A prospective cohort study

**DOI:** 10.1371/journal.pone.0285923

**Published:** 2023-06-02

**Authors:** Siena Molina, Ana Martinez-Urrea, Komal Malik, Ginebra Libori, Helena Monzon, Pablo Martínez-Camblor, Pere Almagro

**Affiliations:** 1 Multimorbidity Unit, Internal Medicine Service, University Hospital Mutua de Terrassa, University of Barcelona, Terrassa, Spain; 2 Department of Anesthesiology, Dartmouth-Hitchcock Medical Center, Lebanon, New Hampshire, United States of America; 3 Faculty of Health Sciences, Universidad Autonoma de Chile, Providencia, Chile; University of Copenhagen: Kobenhavns Universitet, DENMARK

## Abstract

**Background:**

Data about long-term prognosis after hospitalisation of elderly multimorbid patients remains scarce.

**Objectives:**

Evaluate medium and long-term prognosis in hospitalised patients older than 75 years of age with multimorbidity. Explore the impact of gender, age, frailty, physical dependence, and chronic diseases on mortality over a seven-year period.

**Methods:**

We included prospectively all patients hospitalised for medical reasons over 75 years of age with two or more chronic illnesses in a specialised ward. Data on chronic diseases were collected using the Charlson comorbidity index and a questionnaire for disorders not included in this index. Demographic characteristics, Clinical Frailty Scale, Barthel index, and complications during hospitalisation were collected.

**Results:**

514 patients (46% males) with a mean age of 85 (± 5) years were included. The median follow-up was 755 days (interquartile range 25–75%: 76–1,342). Mortality ranged from 44% to 68%, 82% and 91% at one, three, five, and seven years. At inclusion, men were slightly younger and with lower levels of physical impairment. Nevertheless, in the multivariate analysis, men had higher mortality (p<0.001; H.R.:1.43; 95% C.I.95%:1.16–1.75). Age, Clinical Frailty Scale, Barthel, and Charlson indexes were significant predictors in the univariate and multivariate analysis (all p<0.001). Dementia and neoplastic diseases were statistically significant in the unadjusted but not the adjusted model. In a cluster analysis, three patterns of patients were identified, with increasing significant mortality differences between them (p<0.001; H.R.:1.67; 95% CI: 1.49–1.88).

**Conclusions:**

In our cohort, individual diseases had a limited predictive prognostic capacity, while the combination of chronic illness, frailty, and physical dependence were independent predictors of survival.

## Introduction

Global life expectancy has increased progressively in recent decades, reaching 80 years in some developed countries. According to World Health Organization data, the number of persons aged 80 years or older nearly tripled between 1990 and 2019. Projections are for an increase of around 200% by the year 2050, reaching 426 million people [1]. The ageing population is linked to a significant increase in the prevalence of elderly patients with several chronic diseases [2]. Given that the concurrence of chronic pathologic conditions in the same subject impairs symptoms, quality of life, and prognosis, multimorbidity seems a more appropriate term than the classic concept of comorbidity. While comorbidity considers the existence of a primary illness and examines the prevalence and impact of other chronic diseases associated with this central disease, multimorbidity is a broader concept that encompasses the influence of any combination of two or more chronic diseases in the same subject [3, 4].

In Europe, the prevalence of multimorbidity in people over 85 has reached figures of 90% [2, 5]. In older adults, multimorbidity is related to frailty, functional dependence, polypharmacy, hospitalizations, and survival [6, 7]. Nevertheless, some authors consider that the current definition of multimorbidity—based exclusively on the presence of two or more chronic pathologies—is excessively wide. The impact of the association of two or more chronic diseases on an individual subject differs between the different effects of the combination of several chronic disorders and their severity. Additionally, the current definition of multimorbidity does not contemplate the relationship between these chronic diseases and frailty, physical impairment, or prognosis [8, 9]. Although related, multimorbidity, functional dependence, and frailty have an additive and independent effect on the evolution of these patients [10, 11].

For this reason, some authors have proposed alternative definitions of multimorbidity, considering the impact of the combination of several chronic diseases and their effects on disability, quality of life, and care needs [8]. A subgroup of these patients is characterised by frail elders with repeated hospitalisations and increased ambulatory needs that require more complex health care [12, 13].

The National Institute for Health and Care Excellence (NICE) guidelines highlighted the need for prospective studies on patients with well-defined multimorbidity criteria to evaluate their prognosis [14]. Although previous publications have analysed the prognosis of multimorbidity in elderly patients, data on medium- and long-term mortality in this population are lacking [10, 15–17].

Our main objective was to explore the medium- and long-term evolution of older patients with well-defined multimorbidity criteria after hospital discharge, alongside the impact of gender, frailty, functional dependence, and the combination of chronic diseases on mortality.

## Methods

In this prospective cohort study, all patients admitted to a hospital medical ward specialised in the care of multimorbidity patients in the University Hospital Mutua de Terrassa, from September 1, 2015, to December 31, 2016, were evaluated. The unit is dedicated to preventing, detecting, and treating complications, including geriatric syndromes of hospitalised patients with acute medical illness or decompensation of chronic pathologies, preserving physical capacity, individualising the management plan, and coordinating the hospital discharge with outpatient care units.

The present study included hospitalised patients older than 75 with two or more chronic diseases. Comorbidities were measured using the Charlson comorbidity index, which comprises 19 chronic diseases graded by severity [18]. This index was widely used to measure the prognosis of chronic disorders and was extensively validated in different populations, including hospitalised older adults [19]. The Charlson comorbidity index can be expressed with and without age adjustment (one point is added for each decade after 50 years). Our study described both scores, although the non-adjusted index was used in the Cox regression analysis since age is already included in the model. Other relevant comorbid conditions not included in the Charlson index were collected using a standardised previously published questionnaire [20]. All chronic diseases evaluated in both questionnaires are detailed in S1 Table in S1 File. Only the first hospitalisation during the inclusion period was analysed, excluding subsequent readmissions. Since our objective was to explore medium- and long-term prognosis, deceased patients during index admission were also excluded.

During the stay, medical, social, and treatment variables were collected. Baseline functional dependence for ten basic daily living activities was evaluated with the Barthel index. This index measures performance and patient independence concerning self-care, sphincter management, transfers and locomotion. Possible punctuations range between 0 to 100 points, where scores less than 20 points are interpreted as total dependence and greater than 80 as functional independence [21]. Cognitive status was measured using the Pfeiffer Short Portable Mental Status Questionnaire (SPMSQ). The SPMSQ evaluate the number of errors on ten questions, including orientation, memory and attention. Thus, individual cognitive scores ranged from 0 to 10 errors, with lower values indicating better cognitive performance [22].

Frailty was classified according to the Clinical Frailty Score, a measure of frailty based on clinical judgment, in nine stages (one: very robust to eight, very severe frailty). An additional category (9: approaching the end of life) refers to people with a life expectancy < 6 months who are not otherwise living with severe frailty. Since this category is difficult to define in our population and following the recommendations, categories 8 and 9 were grouped [23, 24] (S2 Table in S1 File).

Both the Barthel index and Clinical Frailty scale were assessed using health status two weeks before admission. Delirium was diagnosed using the confusion assessment method, and a speech therapist evaluated dysphagia [25]. Additionally, patients were classified in a non-exclusive form into the most clinically relevant disease patterns:

1. Heart diseases (ischaemic heart disease, heart failure, or atrial fibrillation).
2. Chronic respiratory diseases (chronic obstructive pulmonary disease, asthma, or interstitial pulmonary disease).
3. Psychological diseases (previous diagnosis and treatment of depression or anxiety).
4. Diabetes mellitus with neuropathy or retinopathy.
5. Other metabolic diseases (previous diagnosis and treatment of arterial hypertension, dyslipidemia, or obesity with a body mass index ≥30).
6. Dementia, at least moderate (SPMSQ >5 points or Mini-Mental State Examination <20 points).
7. Chronic kidney disease (glomerular filtration <60).
8. Neurological motor disorders.
9. Neoplasm without curative treatment
10. Osteoarticular diseases with secondary impaired mobility (Barthel ≤60).

Patient data are shared through an electronic medical record with the primary care physician, nurses, and specific outpatient units focused on the care of chronic complex patients. The follow-up for mortality was done through telephone calls, contact with the primary care physician, and review of electronic medical records. The follow-up was completed in June 2022.

### Statistical analysis

Qualitative variables were expressed as absolute frequencies and percentages. Quantitative variables were summarised as mean ± standard deviation (SD) or median and interquartile range 25%-75% (IQR: 25–75%) for skewed distributions. Comparison among location parameters was made with the Welch or with the non-parametric Mann-Whitney U test, appropriately. The Chi-2 test or the Fisher exact test was used to compare proportions. Pearson correlation coefficients were used for measuring the linear agreement between continuous variables. The day of hospital discharge was considered the starting point, and those who did not suffer the event (dead) during the follow-up period were considered censored at the end of the study. Kaplan-Meier estimations and their respective pointwise 95% CI were used for approximating survival curves. Associations between each covariate and the time-dependent outcome were summarised through the hazard ratios (HR) computed from proportional hazard (PH) Cox regression models. Their respective 95% CI are also provided. Results from multivariate PH Cox regression models were also reported. In the multivariate model, we included all statistically (p-value below 0.05 in the univariate model) or clinical (sex) relevant variables. We study the collinearity of the model through the coefficient correlations and the robustness of the models by removing highly correlated variables. Notice that, in this study, we use HRs as association measures, which should be interpreted cautiously. Following Stensrud and Hernan, we have not checked the proportional hazard assumption [26].

Finally, to explore the latent groups of patients based on predictive scores, we used a hierarchical unsupervised learning algorithm based on the combination of age, non-stratified Charlson index, Clinical Frailty Scale and Barthel index. We used the Euclidean distance to construct a Dendrogram. The groups’ numbers were determined after visual inspection and described appropriately.

We did not do a previous sample size computation, and this study includes all patients satisfying inclusion criteria during the enrollment period. Based on the finally obtained numbers, with 500 patients and an overall event percentage of 90%, at the usual nominal level of 5%, we are able to detect HRs above 1.35 with a power of 80% in risk factors present in at least, 25% of the population.

A supporting information file details the STROBE checklist for cross-sectional studies [27] (S3 Table in S1 File). Statistical significance was established at p-values below 0.05. Analysis was performed with MedCalc software version 20.113 (MedCalc Software Ltd, Ostend, Belgium).

### Ethical considerations

Informed consent was obtained from all the patients or their caregivers. The signature was always made in the presence of the researcher and the patient. The Ethics and Clinical Trials Committee of the University Hospital Mutua de Terrassa approved the study.

## Results

**Fig 1:**
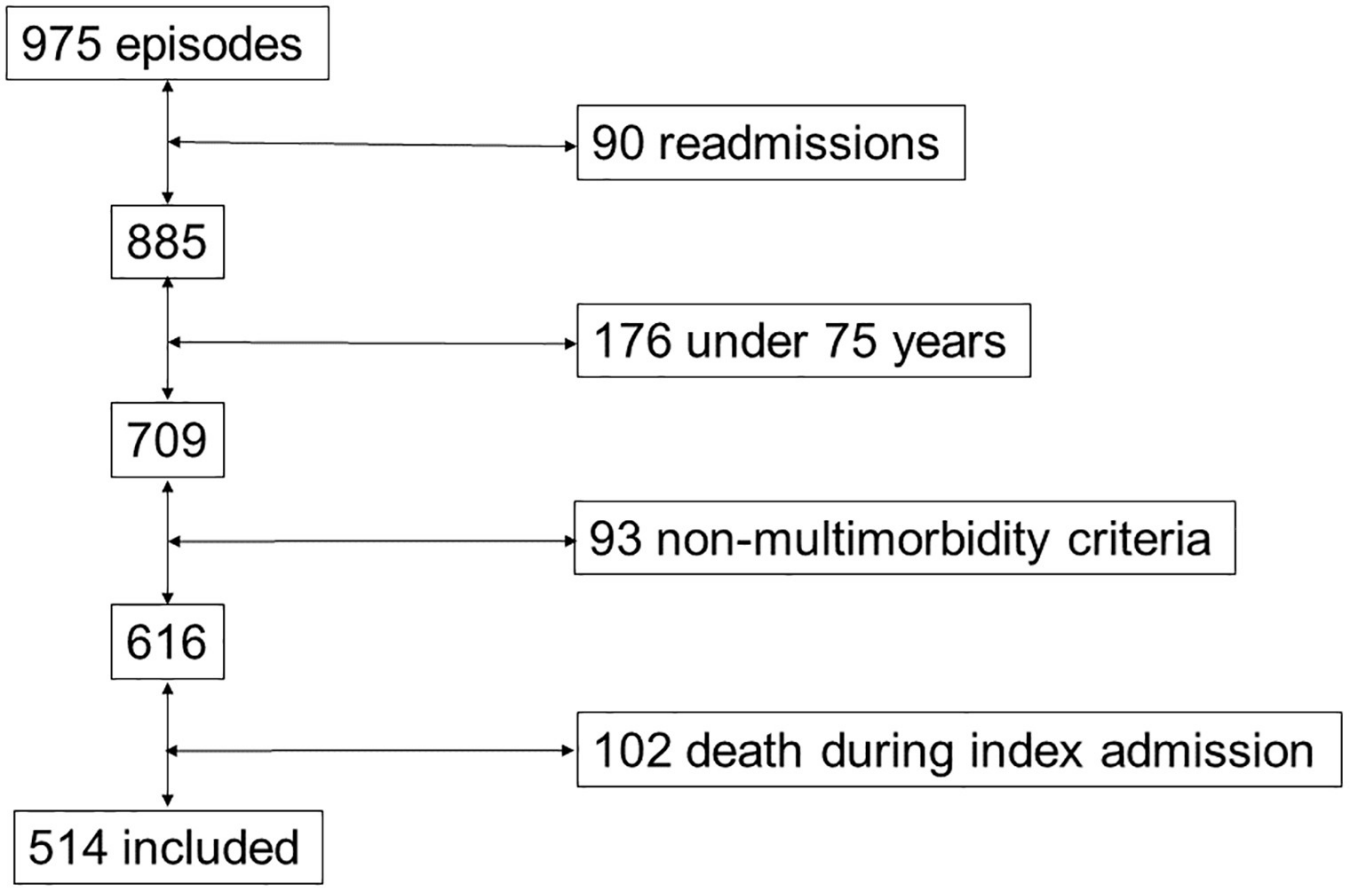
Flowchart of participants.

Of a total of 975 admissions analyzed, 90 were readmissions during the inclusion period, and 269 patients did not meet our age or multimorbidity inclusion criteria. Of the remaining 616 subjects, 102 died during the index admission and were excluded from follow-up. These deceased patients during the first hospitalization were excluded since the follow-up for mortality was carried out from the day of hospital discharge. These patients were older, with more chronic diseases measured by non-age adjusted Charlson index and more significant functional impairment in the Barthel scale, without gender differences. (S4 Table in S1 File) Finally, we included 514 patients with a mean age of 85.3 years with a standard deviation (±S.D.) of 5.3 years. Of these, 234 (45.5%) were men and 280 (54.5%) women. ""Fig 1"" The main characteristics of the total population and the population stratified by gender are detailed in Table 1.

**Table 1:** Differences by gender in the studied population.

	TOTAL (n = 514)	MEN (n = 234)	WOMEN (n = 280)	p-value
Quantitative variables				
Age, years	85.28 (5.32)	84.65 (5.36)	85.80 (5.25)	0.015
Charlson non-adjusted*	4 (3–6)	4 (3–6)	4 (3–6)	0.027
Charlson age-adjusted*	8 (7–10)	9 (7–10)	8 (7–10)	0.053
Barthel*	50 (15–80)	65 (29–90)	40 (10–65)	<0.001
SPMSQ *	3 (0–9)	2 (0–6)	6 (1–9)	<0.001
Number of domiciliary drugs*	9 (6–11)	8 (-11)	9 (6–11)	0.215
Hospital stay (days)*	9 (6–13)	9 (6–13)	8 (6–12)	0.126
Hospitalizations in the previous year*	1(0–3)	1 (0–3)	1 (0–3)	0.080
Clinical frailty score	6 (5–8)	6 (4–8)	7 (6–8)	<0.001
Qualitative variables				
Coexistence				
Alone	83 (16.1%)	38 (16.2%)	45 (16.1%)	0.022
Family	325 (63.2%)	158 (67.5%)	167 (59.5%)	
Professional caregivers or nursing home	101 (19.6%)	34 (14.5%)	67 (23.9%)	
Delirium during admission	280 (55%)	112 (48%)	168 (60%)	0.006
Dysphagia during admission	250 (49%)	100 (43%)%	170 (61%)	0.037
Others	5 (1%)	4 (1.7%)	1 (0.4%)	
One-month readmissions after discharge^¥^	96 (18.7%)	47 (20.1%	19 (17.5%)	0.262
Chronic diseases				
Hearth failure	286 (55.6%)	120 (51.3%)	166 (59.3%)	0.075
Ischaemic heart disease	113 (22%)	64 (27.4%)	49 (17.5%)	0.008
Autoimmune diseases	48 (9.3%)	19 (8.1%)	29 (10.4%)	0.448
Chronic kidney failure	223 (43.4%)	113 (48.3%)	110 (39.3%)	0.049
Chronic respiratory diseases	231 (44.9%)	123 (52.6%)	108 (38.6%)	0.002
Inflammatory bowel disease	9 (1.8%)	3 (1.3%)	6 (2.1%)	0.520
Symptomatic liver disease	16 (3.1%)	7 (3%)	9 (3.2%)	1.000
Cerebrovascular attack	110 (21.4%)	49 (20.9%)	61 (21.8%)	0.830
Motor neurological disease	49 (9.5%)	21 (9%)	28 (10%)	0.764
Cognitive impairment	222 (43.2%)	88 (37.6%)	134 (47.9%)	0.020
Symptomatic peripheral artery disease	52 (10.1%)	34 (14.5%)	18 (6.4%)	0.003
Diabetes mellitus with complications	117 (22.8%)	53 (22.6%)	64 (22.9%)	1.000
Chronic anaemia (Hb < 10 g/dl)	79 (15.4%)	32 (13.7%)	47 (16.8%)	0.390
Solid or haematologic neoplasia	60 (11.7%)	35 (15%)	25 (8.9%)	0.039
Other diseases				
Arterial hypertension requiring treatment	392 (76.3%)	175 (74.8%)	217 (77.5%)	0.532
Depression requiring treatment	126 (24.5%)	37 (15.8%)	89 (31.8%)	<0.001
Anxiety requiring treatment	123 (23.9%)	39 (16.7%)	84 (30%)	<0.001
Dyslipidemia requiring treatment	117 (22.9%)	53 (22.7%)	64 (22.9%)	1.000
Atrial fibrillation	171 (33.3%	80 (34.2%)	91 (32.5%)	0.708
Apnoea-hypopnoea obstructive syndrome	24 (4.7%)	13 (5.6%)	11 (3.9%)	0.408
Osteoarticular diseases with Barthel <60	116 (22%)	29 (12.4%)	87 (31.1%)	<0.001

* Non-normal distribution (median IQR 25.75%).

^¥^Excluding deceased patients without readmission

**Fig 2:**
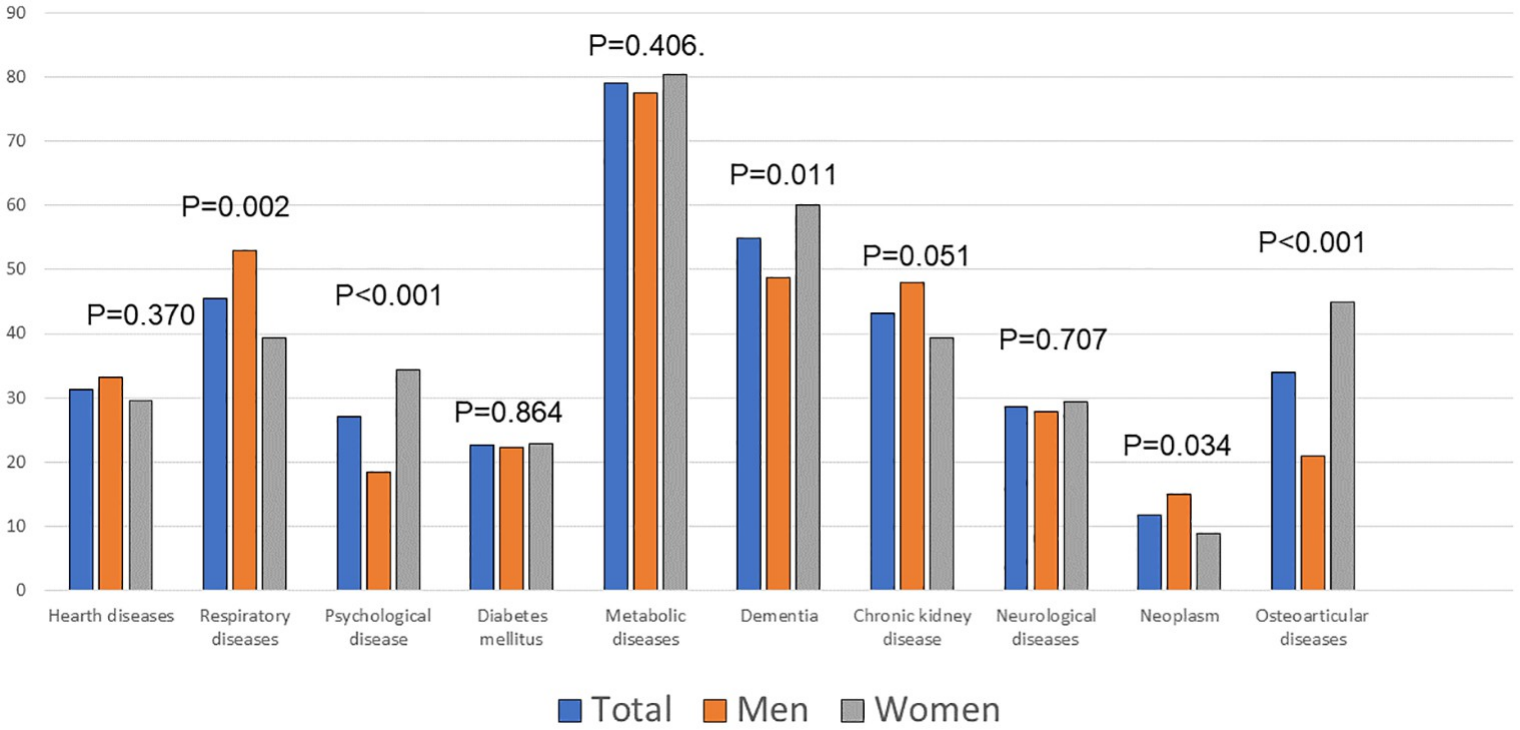
Gender differences by grouped chronic diseases.

Women had more functional dependence for basic daily living activities measured with the Barthel index and higher scores on the Clinical Frailty Scale and Charlson index. We did not observe differences by gender concerning the number of hospitalisations in the previous year, number of domiciliary chronic treatments, or length of stay. Women had a higher prevalence of osteoarticular diseases, anxiety, depression, and dementia. In contrast, men had a higher prevalence of ischemic heart disease, respiratory diseases, and neoplasms. ""Table 1"" A similar pattern was observed in grouped disease analysis. “Fig 2”

### Survival follow-up

The median follow-up was of 485 days (IQR 25–75%: 76–1342), 309 days (IQR 25–75%:51–893) for the deceased patients, and 2,082 days (IQR 25–75%:1,070–2,282) for the 434 censored (415 still alive at the end of the study and 19 were lost in follow-up). The follow-up represented a total of 1063 patients-year. They were classified as censored (alive) on the last day on which they were recorded as survivors in the clinical history of a medical visit or procedure. The median follow-up for the 19 patients without a complete follow-up was 574 days (IQR 25–75%: 93–806).

**Fig 3:**
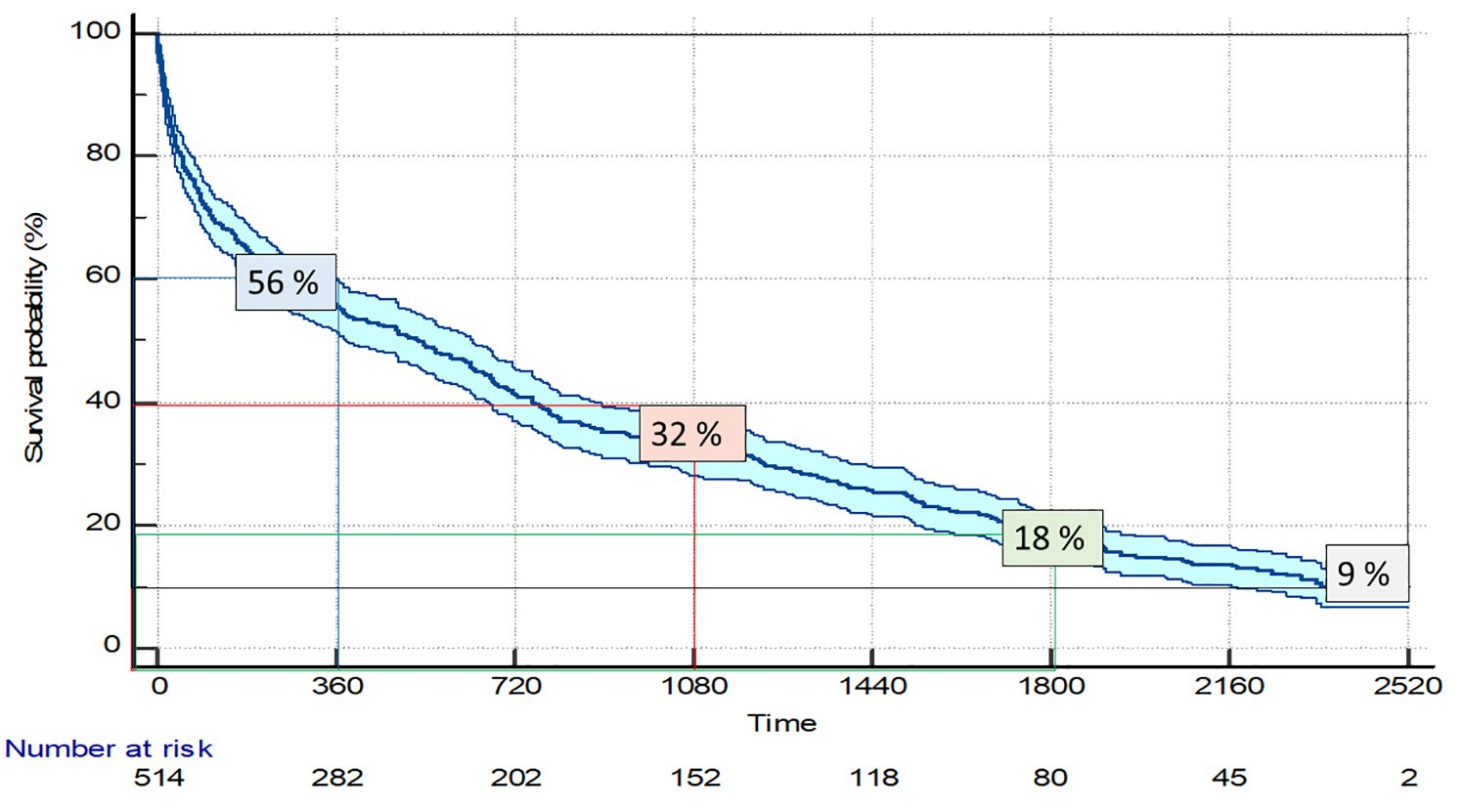
Survival Kaplan-Meier curves with percentages and the 95% confidence interval for the total population.

Survival at the end of follow-up is detailed in Fig 3. “Fig 3” Mortality increased from 44% to 68%, 82%, and 91% at one, three, five, and seven years, respectively.

### Survival follow-up

In the non-adjusted analysis, Age, Clinical Frailty Scale, Barthel, and Charlson index were significantly associated with mortality. Highlight that one additional point in the Clinical Frailty Scale (0.53 standard deviations) represents an increase of 31% in the risk of death (95% CI around 20% and 40%, approximately). In contrast, in the study of chronic diseases, only dementia, chronic kidney disease, and neoplastic diseases were significant in this analysis. The presence of Diabetes mellitus increased the mortality risk by 20%, but it did not reach a significant p-value (p = 0.097) ““Table 2””) (see also S1 Fig in File).

**Table 2:** Hazard ratios (HR) and 95% CI derived from the proportional hazard Cox regression models.

Survival (Univariate model)			
	PH Cox-regression		
	p-value	HR.	95% CI.
Age, year	<0.001	1.04	1.02–1.06
Gender (men)	0.934	1.01	0.83–1.22
Scales			
Barthel	<0.001	0.99	0.99–0.99
Charlson non-adjusted	<0.001	1.05	1.03–1.07
Clinical Frailty scale	<0.001	1.31	1.23–1.39
Chronic diseases			
Cardiac diseases	0.622	0.95	0.78–1.16
Respiratory diseases	0.214	0.89	0.73–1.07
Psychological diseases	0.818	0.98	0.79–1.21
Diabetes mellitus	0.097	1.21	0.97–1.50
Metabolic diseases	0.178	0.18	0.93–1.50
Dementia	0.017	1.26	1.04–1.52
Chronic kidney failure	0.019	1.26	1.04–1.52
Neurological diseases	0.350	1.11	0.90–1.36
Neoplastic diseases	<0.001	1.66	1.24–2.19
Osteoarticular diseases	0.283	0.90	0.73–1.10
Survival (Multivariate model)			
	PH Cox-regression models analysis		
	p-value	HR.	95% CI.
Age, years	<0.001	1.04	1.02–1.05
Gender (men)	0.001	1.41	1.15–1.73
Barthel	0.006	1.01	1.01–1.02
Charlson non-adjusted	<0.001	1.14	1.09–1.19
Clinical Frailty scale	<0.001	1.74	1.44–2.10
Dementia	0.143	1.18	0.94–1.49
Neoplastic diseases	0.079	0.79	0.57–1.0.3
Chronic kidney failure	0.815	0.98	0.80–1.20

HR: Hazard ratio. HR 95% CI: 95% confidence interval for HR.

In the multivariate model, all significant variables in univariate analysis and gender were included. In this model, Gender, Age, Clinical Frailty Scale, Barthel, and Charlson index were independent predictors of mortality. Gender reached statistical significance in this model (HR: 1.41; CI 95%: 1.15–1.73;p = 0.001), while dementia, chronic kidney failure and neoplastic diseases were non-significant. (S2 Fig in S1 File). HR associated with the Clinical Frailty scale increased from 1.31 in the unadjusted to 1.74 in the adjusted model. That is, the relevance of the Clinical Frailty scale increase when the rest of the covariates in the model (including age, gender, and comorbidity index, among others) keep constant. The correlation among the coefficients in the model was low or moderate (values lower than 0.5) except for the correlation between the Clinical Frailty Scale and the Barthel index (0.90). The Pearson correlation coefficient between these variables was -0.94 (95% CI -0.95 to -0.93, p<0.001) (S5 Table in S1 File). For this reason, we performed two additional exploratory models, excluding the Barthel index or Clinical Frailty Scale, which showed similar results (S6 Table in S1 File).

#### Latent risk groups

**Fig 4:**
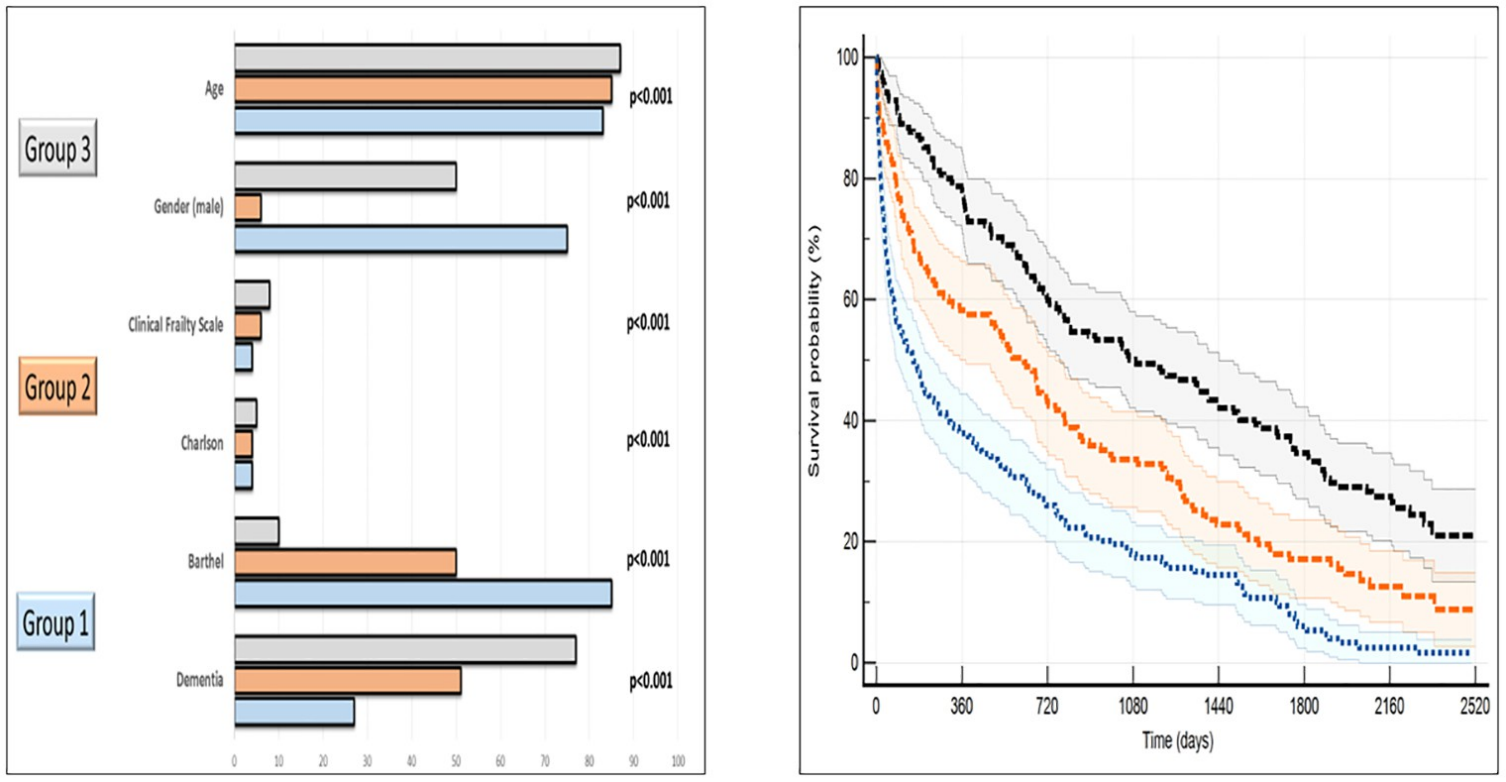
A: Differences by clusters. B: Kaplan-Meier curves and 95% confidence intervals by clusters.

After visual inspection of the dendrogram, three clusters of patients were identified, with 155 (30.2%), 141 (27.6%), and 214 (42.2%) patients. The first (moderate risk) were predominantly males (75%), with younger age, less physical dependence, and lower scores on the Clinical Frailty scale. The second group (severe risk) had a high percentage of women (94%), with higher scores on the Clinical Frailty scale and greater functional dependence. Finally, the patients in the third group (very severe risk) were older, with severe physical impairment, frailty, and higher Charlson scores. In this group, the gender proportion was more balanced. Dementia prevalence and motor neurological diseases increased between the first, second, and third groups. Follow-up mortality significantly differed between groups (p<0.001; H.R.:1.67; 95% CI: 1.49–1.88). ""Table 3"" “Fig 4”

**Table 3:** Cluster analysis.

	Group 1 (n = 155)	Group 2 (n = 141)	Group 3 (n = 214)	p-value
Gender				
Men	74.8%	6.3%	50.2%	<0.001
Women	25.2%	93.7%	49.8%	
Age	82.6 (4.92)	85.10 (5.48)	87.14 (4.77)	<0.001
Scales				
Barthel*	85 (70–100)	50 (44–65)	10 (0–30)	<0.001
Charlson non-age-adjusted*	4 (3–5)	4 (3–7)	5 (3–6)	<0.001
Clinical frailty scale*	4 (4–5)	6 (6–7)	8 (8–9)	<0.001
Chronic diseases				
Cardiac diseases	74.2%	73.2%	61.8%	0.015
Respiratory diseases	56.8%	45.8%	37.3%	0.001
Psychological diseases	15.5%	37.3%	28.6%	<0.001
Diabetes mellitus	19.4%	28.9%	20.7%	0.102
Metabolic diseases	80.9%	79.3%	77.8%	0.765
Dementia	26.5%	51.4%	77.4%	<0.001
Chronic kidney disease	45.8%	40.8%	42.9%	0.684
Motor neurological diseases	16.1%	31.7%	35.5%	<0.001
Neoplasm	9.7%	14.1%	11.5%	0.495
Osteoarticular diseases	21.9%	45.8%	35%	<0.001

*Expressed as median and IQR 25%-75%.

## Discussion

Our study shows that hospitalised elderly patients with multimorbidity have high medium- and long-term mortality, ranging between 44%, 68%, and 82% at one, three, and five years, respectively. The prospective design and the extended follow-up allowed us to confirm the prognostic impact of frailty, functional dependence, age, and the combination of several diseases in survival beyond the relevance of individual illness, corroborating the complex process of evaluating prognosis in this population.

The characteristics of the population can explain the elevated mortality observed in our cohort. Nevertheless, this population represents a high percentage of hospitalised patients in geriatrics and internal medicine services [28–31]. The reduced life expectancy suggests that some diagnostic and therapeutic practices should be individualised, especially those focused on medium- and long-term primary prevention.

In our cohort, women were slightly older but with more significant functional impairment and frailty levels. Although gender mortality was similar in the non-adjusted analysis, women had better survival after adjustment in the multivariate model, confirming the male-female health-survival paradox. This paradox refers to the greater life expectancy in women being penalised by an increased burden of disabling physical impairment and functional dependence. In other words, males have higher mortality after hospital discharge for similar levels of frailty, physical dependence, age, and chronic diseases. This paradox has been well demonstrated in epidemiological studies, but in hospitalised patients with multimorbidity, its presence is controversial, given the low-term follow-up of the available studies [32–34].

Clinical frailty is a state characterised by a limited physical or cognitive functional reserve that reduces the capacity to maintain or restore homoeostasis after a stressor event, and it is a consequence of the cumulative ageing decline in physiological systems [24]. Frailty and disability are usually interrelated but are different concepts. A frail older person without an established disability can develop physical dependence after a stressor episode (e.g. hospitalization) [35]. For the same level of frailty, the more severe events have a greater risk of disability, and vice versa; patients with greater previous functional reserve or lower frailty can better recover from the impairment produced by an event of similar severity [36]. Frailty is also related to complications during hospitalization, such as delirium, dysphagia, deconditioning at discharge, risk of readmissions and mortality [36–42]. To date, multiple scales and tests have been developed to explore frailty in different populations [43–45]. In our case, we used the Clinical Frailty Scale 2.0, which grades frailty into nine categories (1 very robust to 9 terminally ill). The Clinical Frailty Scale has been extensively validated in different populations of elderly patients [46–50]. It is not a questionnaire; its classification is based on clinical judgment, so it has an inherent component of subjectivity. However, interobserver concordance after minimal training can be considered reasonable [51, 52].

In our multivariate analysis, frailty, age, comorbidities, physical dependence for basic activities of daily living, and gender were independent prognostic predictors. In contrast, none of the chronic diseases was a significant independent predictor of mortality in the adjusted analysis, suggesting that in this population, the combination of chronic conditions and frailty is more relevant than the prognosis of an individual illness. These results are in concordance with several recent studies that have shown a prognostic interplay between these variables and short-term evolution in patients with multimorbidity [8, 10, 11, 19, 20]. Nevertheless, to our knowledge, no previous studies have explored this in medium- and long-term follow-up.

Finally, we performed a cluster analysis including relevant prognostic variables. In this approach, three patterns of severity were identified. The population was predominantly male in the first of these, while 94% were females in the second, without gender differences in the third group. Age, Barthel, Charlson, Clinical Frailty scale, dementia, and motor neurological diseases consistently increased between the first and third patterns. Mortality also increased in a significant way between the three clusters.

Our study has several strengths and limitations. It is a prospective study with well-defined criteria and extensive follow-up. However, the cohort was recruited in a single centre, with a specialised unit focused on the care of these patients, so perhaps the results cannot be extrapolated to other populations. However, the number of included patients was considerable, and their characteristics were similar to those observed in multicenter studies [28, 29, 40, 53, 54].

In conclusion, our data confirm the high medium- and long-term mortality in elderly hospitalised patients with multimorbidity. Our study reinforces that the classical prognostic evaluation based on a single disease in this population is less relevant than the combination of chronic pathologies, frailty, and functional dependence. The reduced life expectancy suggests that some diagnostic and therapeutic practices should be individualised, especially those focused on medium- and long-term primary prevention. Finally, our data confirm the male-female health-survival paradox.

## Supporting information

S1 File (DOCX)
