# Supplementary material for: Medium and long-term prognosis in hospitalised older adults with multimorbidity. A prospective cohort study
Source: PLoS One. 2023 Jun 2;18(6):e0285923. doi: 10.1371/journal.pone.0285923 (PMC10237495; doi:10.1371/journal.pone.0285923)
Supplement: S1 File — (DOCX) [file pone.0285923.s001.docx]

**Supplementary material**

Medium and long-term prognosis in hospitalised older adults with multimorbidity. A prospective study.

**Table S1**

| **Chronic diseases included in the Charlson index** |
| --- |
| Ischemic heart disease |
| Heart failure |
| Peripheral vascular disease |
| Cerebrovascular disease |
| Dementia |
| Chronic respiratory disease |
| Connective tissue disease |
| Ulcer disease |
| Liver disease (mild) |
| Diabetes M without organ damage |
| Kidney Disease (creatinine<3) |
| Hemiplegia |
| Kidney Disease (creatinine>3) |
| Diabetes Mellitus with organ damage. |
| Malignant solid tumour |
| Leukemia |
| Lymphoma |
| Liver Disease (severe) |
| Malignant solid tumour with metastases |
| AIDS |
| **Chronic diseases not included in the Charlson index** |
| Arterial systemic hypertension |
| Osteoporosis |
| Depression |
| Anxiety |
| Dyslipidemia |
| Apnoea-hypoapnoea obstructive syndrome |
| Atrial fibrillation |
| Sick sinus disease |
| Atrioventricular block |
| Iron-deficiency anaemia |
| Other anaemias |
| Obesity |
| Thromboembolic disease |

Table S2

Clinical Frailty score

|  |  |  |
| --- | --- | --- |
| 1 | Very fit | Robust, active |
| 2 | Fit | No active disease symptoms |
| 3 | Managing well | Medical problems well controlled |
| 4 | Very mild frailty | Vulnerable. Symptoms limit activities |
| 5 | Mild frailty | Needs help with instrumental activities |
| 6 | Moderate frailty | Needs help with basic activities of daily life |
| 7 | Severe frailty | Completely dependent, life expectancy > 6 months |
| 8 | Very severe frailty | Completely dependent, approaching end of life |
| 9 | Terminally ill | Approaching the end of life. This category applies to people with a life expectancy < 6 months, who are not otherwise living with severe frailty. |

Table S3

STROBE Statement—Checklist of items that should be included in reports of ***cohort studies***

|  | **Item No** | **Recommendation** |
| --- | --- | --- |
| **Title and abstract** | 1 | (*a*) Indicate the study’s design with a commonly used term in the title or the abstract (lines 1-28) |
|  |  | (*b*) Provide in the abstract an informative and balanced summary of what was done and what was found (lines 1-28) |
| **Introduction** | | |
| Background/rationale | 2 | Explain the scientific background and rationale for the investigation being reported (lines 56-92) |
| Objectives | 3 | State specific objectives, including any prespecified hypotheses (lines 93-96) |
| **Methods** | | |
| Study design | 4 | Present key elements of study design early in the paper (line 101) |
| Setting | 5 | Describe the setting, locations, and relevant dates, including periods of recruitment, exposure, follow-up, and data collection (lines 101-107) |
| Participants | 6 | (*a*) Give the eligibility criteria, and the sources and methods of selection of participants. Describe methods of follow-up (lines 108-138) |
|  |  | (*b*) For matched studies, give matching criteria and number of exposed and unexposed. Non-applicable |
| Variables | 7 | Clearly define all outcomes, exposures, predictors, potential confounders, and effect modifiers. Give diagnostic criteria, if applicable (lines 108-138) |
| Data sources/ measurement | 8* | For each variable of interest, give sources of data and details of methods of assessment (measurement). Describe comparability of assessment methods if there is more than one group (ñines 108-138) |
| Bias | 9 | Describe any efforts to address potential sources of bias (lines 152-157) |
| Study size | 10 | Explain how the study size was arrived at (non-applicable) |
| Quantitative variables | 11 | Explain how quantitative variables were handled in the analyses. If applicable, describe which groupings were chosen and why (lines 145-148) |
| Statistical methods | 12 | (*a*) Describe all statistical methods, including those used to control for confounding (lines 144-161) |
|  |  | (*b*) Describe any methods used to examine subgroups and interactions (lines 145-161) |
|  |  | (*c*) Explain how missing data were addressed (lines 191-196) |
|  |  | (*d*) If applicable, explain how loss to follow-up was addressed (lines 191-196) |
|  |  | (*e*) Describe any sensitivity analyses (lines 200-210) |
| **Results** | | |
| Participants | 13* | (a) Report numbers of individuals at each stage of study—eg numbers potentially eligible, examined for eligibility, confirmed eligible, included in the study, completing follow-up, and analysed (figure 1) |
|  |  | (b) Give reasons for non-participation at each stage (figure 1) |
|  |  | (c) Consider use of a flow diagram (figure 1) |
| Descriptive data | 14* | (a) Give characteristics of study participants (eg demographic, clinical, social) and information on exposures and potential confounders Table 1) |
|  |  | (b) Indicate number of participants with missing data for each variable of interest (table 1) |
|  |  | (c) Summarise follow-up time (eg, average and total amount) (lines 191-198) |
| Outcome data | 15* | Report numbers of outcome events or summary measures over Time (Figure 2) |
| Main results | 16 | (*a*) Give unadjusted estimates and, if applicable, confounder-adjusted estimates and their precision (eg, 95% confidence interval). Make clear which confounders were adjusted for and why they were included (Table 2) |
|  |  | (*b*) Report category boundaries when continuous variables were categorised (table 2) |
|  |  | (*c*) If relevant, consider translating estimates of relative risk into absolute risk for a meaningful time period (non-applicable) |
| Other analyses | 17 | Report other analyses done—eg analyses of subgroups and interactions, and sensitivity analyses (lines 200-210) Tables supplementary material |
| **Discussion** | | |
| Key results | 18 | Summarise key results with reference to study objectives (lines 228-284) |
| Limitations | 19 | Discuss limitations of the study, taking into account sources of potential bias or imprecision. Discuss both direction and magnitude of any potential bias (lines 285-290) |
| Interpretation | 20 | Give a cautious overall interpretation of results considering objectives, limitations, multiplicity of analyses, results from similar studies, and other relevant evidence (lines 228-284) |
| Generalisability | 21 | Discuss the generalisability (external validity) of the study results (lines 286-288) |
| **Other information** | | |
| Funding | 22 | Give the source of funding and the role of the funders for the present study and, if applicable, for the original study on which the present article is based (non-applicable) |

Table S4

Differences between patients deceased during index admission and discharged alive

|  | Deceased (n=102) | Discharged (n=514) | p-value |
| --- | --- | --- | --- |
| Age | 88 (84-92) | 85 (81-89) | <0.0001 |
| Charlson | 5 (3-7) | 4 (3-7) | 0.007 |
| Barthel | 35 (10-70) | 50 (15-80) | 0.048 |
| Gender (male) | 296 (48%) | 320 (52%) | 0.660 |
| PROFUND | 16 (12-18) | 12 (8-18) | <0.0001 |

Table S5

Correlation matrix of regression coefficients between variables included in the multivariate Cox-regression analysis

| **Correlation Matrix of Regression Coefficients** | | | | | | |
| --- | --- | --- | --- | --- | --- | --- |
|  | Gender | Charlson | Dementia | Neoplastic diseases | Age | Clinical Frailty Scale |
| Charlson | -0.005 |  |  |  |  |  |
| Dementia | -0.032 | -0.068 |  |  |  |  |
| Neoplastic diseases | 0.024 | 0.234 | -0.066 |  |  |  |
| Age | 0.087 | 0.197 | -0.051 | -0.020 |  |  |
| Clinical Frailty Scale | 0.027 | 0.029 | -0.006 | 0.044 | 0.029 |  |
| Barthel | -0.085 | 0.074 | -0.218 | 0.078 | 0.120 | 0.903 |

Table S6

Exploratory models of Cox-regression multivariate analysis including the Clinical Frailty Scale or the Barthel index in the models.

| Exploratory additional model 1 | | | |
| --- | --- | --- | --- |
|  | p-value | H.R. | H.R. 95% C.I. |
| Gender | <0.001 | 1.47 | 1.20-1.81 |
| Charlson | <0.001 | 1.13 | 1.08-1.19 |
| Dementia | 0.003 | 1.41 | 1.13-1.76 |
| Neoplastics diseases | 0.029 | 0.72 | 0.54-0.97 |
| Age | 0.002 | 1.03 | 1.01-1.05 |
| Clinical Frailty Scale | <0.001 | 1.453 | 1.35-1.56 |
| Exploratory additional model 2 | | | |
| Gender | 0.012 | 1.30 | 1.06-1.60 |
| Charlson | <0.001 | 1.13 | 1.08-1.19 |
| Dementia | 0.130 | 1.19 | 0,95-1.50 |
| Neoplastic diseases | 0.0191 | 0.70 | 0.52-0.94 |
| Age | 0.0001 | 1.03 | 1.01-1.05 |
| Barthel | <0.001 | 0.99 | 0.98-0.99 |

Figure S1

Clinical Frailty Score


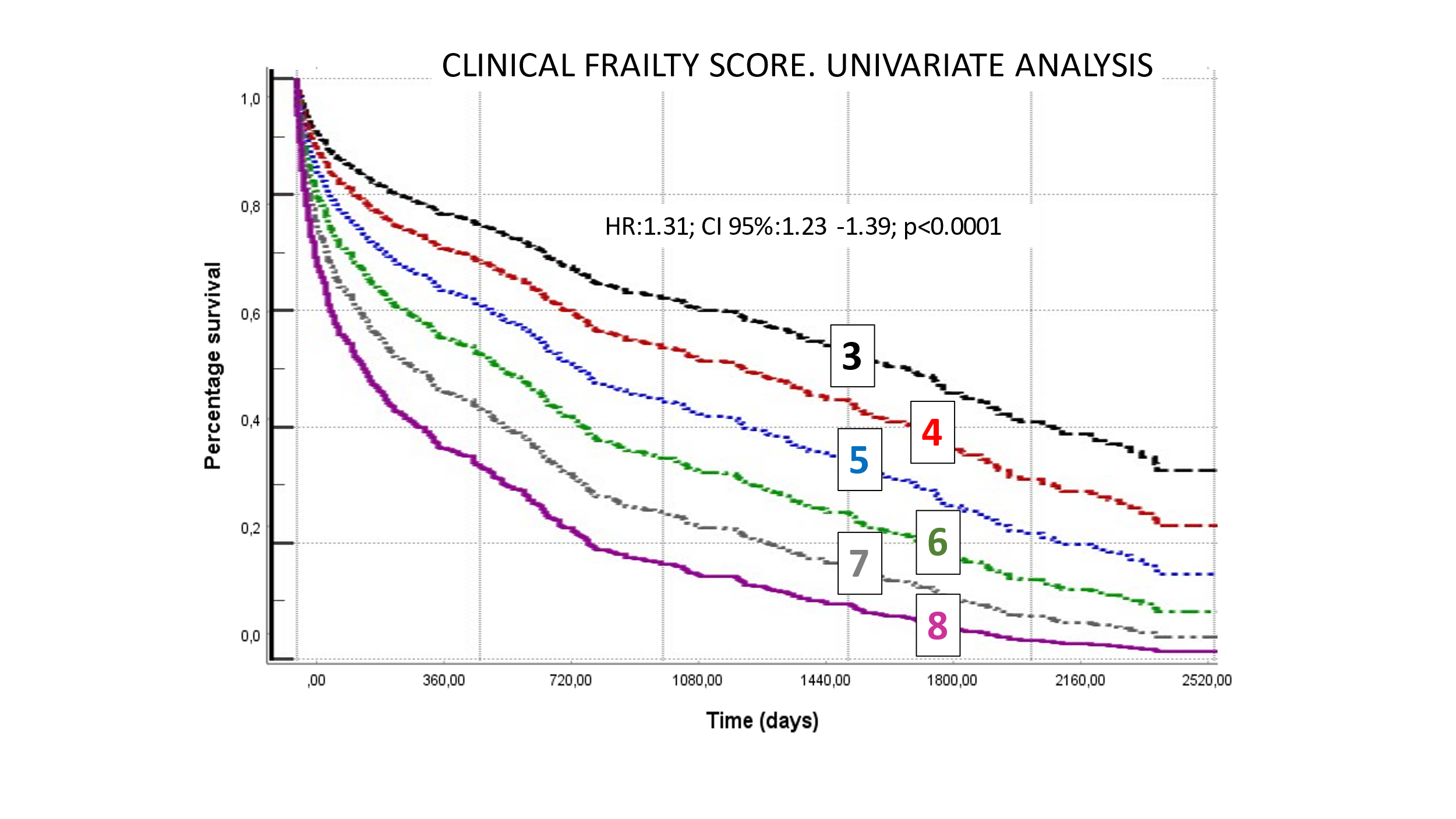


Figure S2

Gender survival


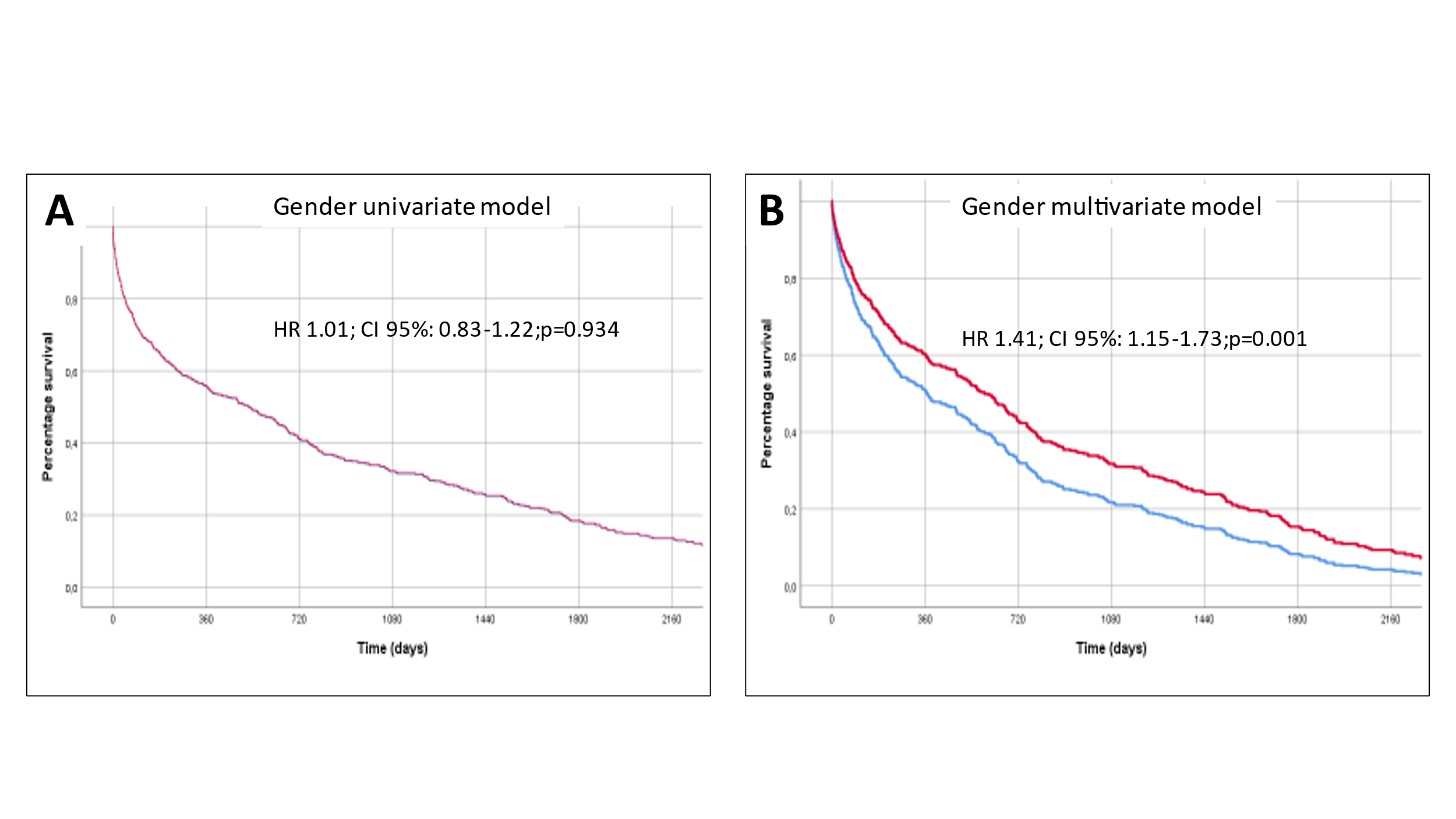


A: Gender univariate analysis. B: Gender multivariate analysis (adjusted by age, Barthel, Charlson, Clinical Frailty Score, dementia, neoplastic diseases and chronic kidney failure.
